# From a Food Safety Prospective: The Role of Earthworms as Food and Feed in Assuring Food Security and in Valuing Food Waste

**DOI:** 10.3390/insects11050293

**Published:** 2020-05-11

**Authors:** Doriana Eurosia Angela Tedesco, Marta Castrica, Aldo Tava, Sara Panseri, Claudia Maria Balzaretti

**Affiliations:** 1 Department of Environmental Science and Policy, Università degli Studi di Milano, via Celoria 2, 20133 Milan, Italy; 2 Department of Health, Animal Science and Food Safety “Carlo Cantoni”, Università degli Studi di Milano, Via Celoria 10, 20133 Milan, Italy; sara.panseri@unimi.it (S.P.); claudia.balzaretti@unimi.it (C.M.B.); 3 CREA Research Centre for Animal Production and Aquaculture, viale Piacenza 29, 26900 Lodi, Italy; aldo.tava@crea.gov.it

**Keywords:** food security, food consumption sustainability, innovative food, alternative sources of protein, food safety

## Abstract

The Sustainable Development Goals are a set of global goals that provide a framework for shared action. These goals also include the reduction of food waste and the definition of sustainable solutions to achieve food security. In this context, the aim of the study was to describe all phases of a pilot earthworm rearing project started in September 2017 and concluded in December 2017, together with a risk analysis carried out in order to evaluate if earthworms can represent a safe and sustainable protein source for human consumption and/or animal nutrition. The conversion rate, that in this study is more appropriately identified as the “waste reduction efficiency,” was also calculated in order to define the extent to which earthworm rearing can contribute to the objective of reducing fruit and vegetable waste (FVW). The results showed that earthworms can bio-convert 3750 kg of FVW in three months producing 1050 kg of compost and 82 kg of fresh earthworms with minimal environmental impact showing good waste reduction efficiency. Moreover, the risk analysis conducted on earthworm rearing highlighted a microbiological hazard after the freeze-drying phase. The critical control point was therefore identified, and, in order to guarantee the total food safety of the finished product, corrective action was taken consisting in the implementation of heat treatment—sterilization at 121 °C for 20 min. The results of microbiological analyses carried out on the earthworm meal after the sterilization treatment showed that the treatment guarantees microbiological safety for the consumer and ensures a balanced approach in relation to two main topics—public health and food-borne diseases. In conclusion, earthworm meal is a concentrate of valuable nutrients useful for human and animal nutrition and can also transform fruit and vegetable waste into a resource.

## 1. Introduction

The Sustainable Development Goals are a set of global goals that provide a framework for shared action to be implemented by all countries and all stakeholders. Specifically, the target of Goal 12 is to “halve per capita global food waste at the retail and consumer level and reduce food losses along production and supply chains” [1], while goal 2 concerns sustainable solutions to end hunger and to achieve food security also through small-scale farmers and sustainable food production systems [2].

Many scientists have focused their research on the analysis of strategic approaches to prevent and minimize food waste in the food supply chain [3,4]. To identify sustainable and safe strategies against food wastage is a priority and, moreover, to turn waste into a resource by valorizing it in the agri-food supply chain is in line with the EU action plan for the Circular Economy [5]. 

In this context, earthworms, like insects, could represent a valuable solution. They can bio-convert fruit and vegetable waste, which this is a sustainable, cost-effective, and ecological approach that contributes to the reduction of food waste [6,7,8,9,10]. Using fruit and vegetable waste as a substrate for the growth of earthworms produces a new high-protein nutrient that can in turn be valued, first for animal nutrition and then for human consumption. The need for new food products is dependent on two specific aspects—(i) the human population is increasing, with more than 821 million people still lacking regular access to adequate food, and, at the same time, (ii) the demand for new protein sources, in particular for animal proteins, which are the most limiting and expensive in terms of resources, is also increasing [11]. Today, the food sector is considering the use of insects in human nutrition [12,13], and, in this context, terrestrial invertebrates such as earthworms used as an alternative source of protein could represent a valid solution, especially if they are fed without land use competition and by reintroducing fruit and vegetable waste into the food supply chain, hence turning waste into a resource [6]. Several authors [14,15] have underlined the nutritional value of earthworms based on their nutrient content: earthworms of *Eisenia foetida* species are an excellent source of biologically valuable protein, micronutrients, minerals, and vitamins in the human diet. *E. foetida* meal has a high protein content in the range of 54.6% to 71% dry matter [14,16,17,18,19,20] and is rich in amino acids considered essential for humans [17]. Earthworms are also rich in fat, with content ranging from 7.3% to 10% of dry matter [17,20]. As reported in Reg. (EC) no. 2015/2283 on novel foods, traditional foods from third countries with a history of safe food use can be considered a valuable source of food nutrients in our food chain. In some parts of Asia, Africa, and South America [16], earthworms have been introduced into the everyday diet and have also been included in the Dictionary of Food Science and Technology [18]. Finally, to ensure a high level of protection of human health, the production of edible terrestrial invertebrates as food should be safe and wholesome and reared and marketed as food following European safety rules for food.

Earthworm meal therefore has interesting nutritional proprieties, but in order to be commercialized as a product for human consumption and/or animal feed, it must be safe for the final consumer. In order to ensure the correct safety standards, the finished food product must undergo assessment to ensure its hygienic-sanitary conformity and the absence of undesirable substances. To guarantee the safety of a finished food product it is necessary to evaluate (i) the microbiologic profile, testing for microorganism indicators of process hygiene and verifying compliance with food safety parameters, and (ii) the chemical profile, for the possible presence of pesticide residues and toxic elements. As regards the latter, a programmed (the National Residues Plan from the transposition of Community Directives 96/22/EC and 96/23/EC) for the surveillance and monitoring of residues of chemical substances that could be harmful to public health in animals and food of animal origin can be adopted at a national level. In a more general perspective, another important aspect is the organoleptic quality of the finished food product that can be verified by sensory analysis through panel tests or by analysis of volatile organic compounds (VOCs) [21].

In this context, the aim of this study was to describe all phases of a pilot earthworm rearing activity developed within the project “Bioconversion of fruit and vegetable waste to earthworm meal as novel food source” funded by FONDAZIONE CARIPLO—Integrated research on industrial biotechnologies 2015 (Project No. 2015-0501). Pilot rearing started in September 2017 and finished in December 2017. During this period, a risk analysis was carried out throughout the entire chain in order to evaluate if earthworm’s meal can represent a safe and sustainable protein source for human consumption and/or animal nutrition. Specifically, microbiological analyses by analyzing presence of the main microorganisms indicating process hygiene and food safety, chemical analyses to verify the absence of residues of chemical substances, and finally VOC analyses to delineate the aromatic profile of earthworm meal, were carried out.

Furthermore, the conversion rate, which in this study is more appropriately identified as the “waste reduction efficiency,” was also calculated in order to define the extent to which earthworm rearing can contribute to the objective of reducing fruit and vegetable waste.

## 2. Materials and Methods

### 2.1. Pilot Earthworm Rearing, Growth Substrate, and Waste Reduction Efficiency

**Figure 1:**
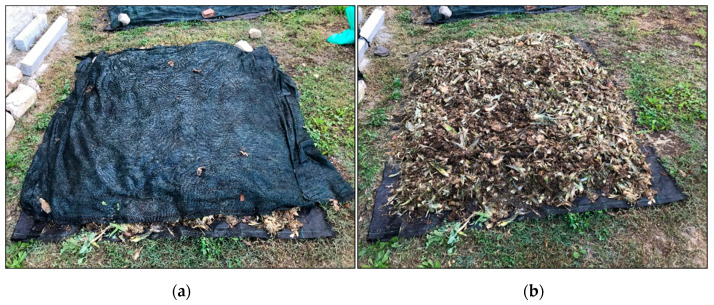
(**a**) Covered earthworm rearing; (**b**) Uncovered earthworm rearing with visible growth substrate.

The present study was approved by the Ethics Committee of the University of Milan, Italy (30.01.17; ethical code number 02/17). The FVW growth substrate was collected weekly from processing industries and consisted mainly of pineapple skins (about 23%), pineapple tufts (about 12%), mango pulp and skins (about 16%), pomegranate skins (about 9%), grape including branches (about 12%), tomato skins (about 10%), kiwi skins (about 9%), and papaya skins (about 9%). The pineapple tufts were ground with a gardening shredder to make them biodegradable by earthworms. The variability of substrate components depended on daily processing industry activity and seasonality. Preliminary tests were carried out to establish optimum earthworm feeding conditions. The waste mixture was chopped and left to rot for a few days before being fed to earthworms, as they can then process it more efficiently. To reach a C:N optimal ratio for earthworm growth the FVW was mixed with straw (10:1) and added three times a month on the top of the production area. The rearing of earthworms (*Eisenia foetida*) was on a pilot scale on a total surface area of 34 m^2^ with a subplot size of 4–5 m^2^ per seven rearing areas (Figure 1). The rearing area was prepared with a non-woven textile sheet to avoid water stagnation and earthworms escaping and covered on the top with a net to avoid predators (Figure 1a). A mix of young non-clitellum and adult clitellate earthworms were added at a density of 1 kg/m^2^ and reared on a feeding substrate of FVW (Figure 1b). The rearing process lasted three months, and during this time, substrates from each growing area were sampled weekly to evaluate moisture, temperature and pH to guarantee favorable conditions to obtain optimum growth, spraying potable water as required to maintain moisture. The conversion rate, in this study more appropriately identified as the “waste reduction efficiency,” was calculated based on the quantity (kg) of FVW that was bio-converted into vermicompost and the yield in fresh earthworms (FE) in three months.

### 2.2. Processing of Earthworms and Transformation Technology into Meal

**Figure 2:**
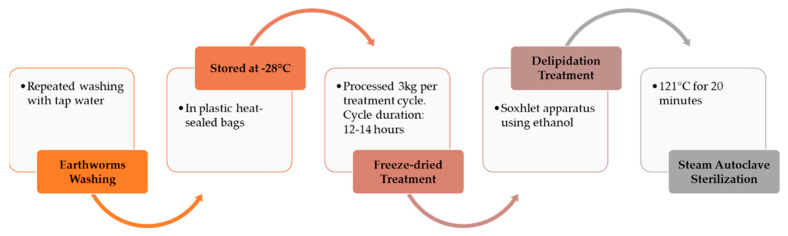
Steps of the transformation process from fresh earthworms to sterilized defatted earthworm meal.

Earthworms from each rearing area were separated mechanically from the vermicompost with the use of a trommel. Once collected, earthworms were subjected to a cleaning procedure. To remove the residual particles of vermicompost and to clean the body surface, they were repeatedly washed with running tap water, left in water until excretion of gut content, washed once more, and placed on paper to remove excess water. Earthworms were packaged in plastic bags, weighed, and stored at −28 °C to allow them to enter quiescence and to kill them. Earthworm meal was obtained by freeze-drying and grinding with a mechanical crusher; the *Eisenia foetida* samples were then defatted in a Soxhlet apparatus using ethanol, a solvent used in the food industry to avoid the presence of residues or derivatives in the final foodstuff or food ingredient [22]. After delipidation treatment, the defatted earthworm meal (DEM) samples underwent a sterilization cycle in a steam autoclave (WVR Vapor Line Autoclave) at 121 °C for 20 min to make the product safe and to stabilize it for subsequent storage at room temperature. All the process steps have been summarized in Figure 2.

### 2.3. Nutritional, Chemical, and Microbiological Analyses

The samples of sterilized DEM (SDEM) were derived from fresh earthworms collected three times over a three-month period (from September 2017 to December 2017) at five different sampling points within the rearing area in order to provide a representative sample. All parameters analysed are reported in Table 1 and all analyses were carried out according to validated or standardized methods. Only the microbiological analyses were carried out both on the FE and the DEM after the freeze-drying and sterilization process in order to highlight critical control points and to avoid microbiological risks and any kind of hazard for human health.

### 2.4. Statistical Analysis

All samples were prepared and analyzed in triplicate. Results are expressed as mean ± standard deviation using SPSS Statistics, version 25 (IBM, SPSS Inc., Chicago, IL, USA). A probability value of *p* < 0.05 was considered statistically significant.

## 3. Results

### 3.1. Substrate of Growth and Waste Reduction Efficiency Results

Table 2 reports the physicochemical characteristics of the growth substrate that were monitored throughout the rearing period. In our experimental conditions in three months of rearing, the bioconversion of 3750 kg of FVW resulted in a production of 82 kg of earthworm biomass (DM 16%) and 1050 kg of vermicompost (the vermicompost process was not discussed in this study). A total of 13.2 kg of earthworm meal was obtained from this reuse of FVW. This would indicate that 6.25 kg of fresh earthworms are required to produce 1 kg of earthworm meal together with the 80 kg of vermicompost. 

### 3.2. Nutritional, Chemical, and Microbiological Results

Table 3 shows the nutrient content of sterilized defatted earthworm meal. The most significant value is in the protein content (73.2% on DM). The results of the mineral analyses showed significant amounts of valuable minerals for nutritional purposes: potassium (4.7 mg g^−1^ on DM) and calcium (4 mg g^−1^ on DM). Moreover, SDEM represents an excellent source of iron (0.3 mg g^−1^ on DM) and iodine (9 µg g^−1^ on DM) toxic heavy metals cadmium and lead were found to be below the detection levels of the methods used.

The results of volatile compounds extracted by using Solid Phase Microextraction and detected by GC/MS are presented in Table 4. The volatile profile showed that ethanol is the main compound identified. The other compounds, especially ketones and aldehydes with hexanal, are present in very low amounts. No residues of antibiotics, pesticides, and mycotoxin were identified in any of the samples analyzed. 

**Figure 3:**
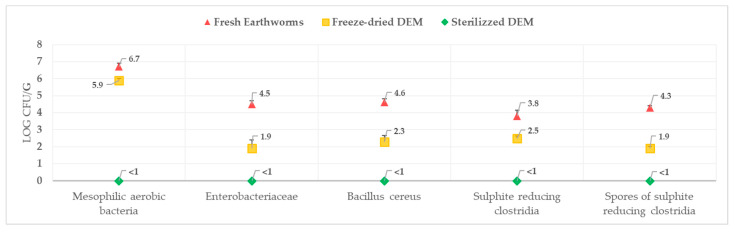
Results of microbiological analyses.

As regards the microbiological analyses, the main results are reported in Figure 3 and were expressed as Log CFU/g; for *Salmonella* spp. and *Listeria monocytogenes*, the results were expressed as presence or absence in 25 g of sample results showed that *E. coli* and coagulase-positive staphylococci were found <1 Log CFU/g in all three types of samples, while for total coliform bacteria the freeze-drying treatment immediately reduced the result to <1 Log CFU/g. For the other microbiological parameters, it is evident that the different phases of technological transformation lead to a decrease in the total bacterial load including sporogenic microorganisms. The reduction is particularly evident for: *Bacillus cereus* (FE = 4.6 Log CFU/g, Freeze-dried DEM = 2.3 Log CFU/g and SDEM ≤ 1 Log CFU/g); sulphite reducing clostridia (FE = 3.8 Log CFU/g, freeze-dried DEM = 2.5 Log CFU/g and SDEM ≤ 1 Log CFU/g); and spores of reducing clostridia (FE = 4.3 Log CFU/g, Freeze-dried DEM = 1.9 Log CFU/g and SDEM ≤ 1 Log CFU/g). Finally, in all three types of samples, *Salmonella* spp. and *Listeria monocytogenes* were found to be absent in 25 g of the sample. 

## 4. Discussion

Turning FVW into earthworm meal could contribute to the production of new sources of protein, without the disadvantages of meat consumption from livestock production, hence addressing future needs for food in a fast-growing population context. This process has the added benefit of organically producing the hummus-like material known as vermicompost, a high-quality fertilizer [26]. The waste reduction efficiency was calculated as reported by Salomone et al. [30]. Starting from 3750 kg FVW used to feed the earthworms, the residue was 1050 kg of vermicompost. Therefore, the efficiency of the waste material reduction was 72%, confirming the positive results of this process. Furthermore, the process converted FWV into edible earthworm meal, a food product with low land use—only the rearing surface, and no-competing feed use. Bearing in mind climate change, the impact of the whole production system per 1 kg of dried earthworm meal and 80 kg of vermicompost was 6.874 kg CO_2 eq_ [6], considerably lower than landfill or incineration waste treatment [31]. This process also brought benefits such as the recovery of FVW as a feeding substrate, earthworm production as a food source with a high nutritional profile, and the availability of vermicompost as an organic fertilizer that allows a reduction in the use of mineral fertilizers in other production systems. For food purposes, earthworms were subjected to a deep washing and vacuum freeze-drying procedure, a useful dehydration method to preserve nutritional quality. Then, the earthworm meal was defatted to obtain a meal rich in protein and other nutrients and to decrease perishability. The results showed that earthworm grown on FVW presented a high protein content, from 62.3% on DM before the defatting process to about 73% on DM in the sterilized defatted sample, similar to percentages reported in other studies [14,16,17,18,19,20]. Moreover, iron and iodine minerals are also well represented. The results of Medina et al. [16] that earthworm meal does not contain significant levels of heavy metals, were confirmed by this study in which toxic heavy metals were below detection levels.

Therefore, the use of SDEM in human nutrition as an ingredient in food preparations can be hypothesized. Although the idea and the benefits are clear, consumer acceptance could be difficult regarding such unknown foodstuffs not traditionally present in the culture of Western countries. Nevertheless, current trends are towards “superfoods” with health benefits and to tackle the issue of potential industrial applications in food preparations, some studies have reported that the best solution for consumer acceptance consists in the invisible inclusion of invertebrates [32,33]. Thanks to new technologies, it is now possible to find an alternative means of nutrient intake by integrating earthworms in food preparations with a specific technological process thus expanding the use of these food sources. new products could be developed such as a hypothetical protein bar including 10 g (10% of 100 g bar) of SDEM among the ingredients, enough to cover about one-sixth of the recommended daily protein requirement for an adult man (70 kg, 19–65 years) [34]. Moreover, the consumption of a protein bar with only 10 g of SDEM contributes to the daily requirement of iron and iodine, respectively, 51% and 60% of the recommended daily intake [35].

No residues of antibiotics, pesticides or mycotoxins were found in our SDEM sample and these results confirm that the meal possesses food safety traits in relation to possible public health risks. Earthworms are able to accumulate some toxic compounds as pesticides and heavy metals stemming from the growth substrate, i.e., soil [36,37]. The non-presence of these types of residues, such as pesticides and heavy metals, is probably due to the fact that the growth substrate that was used in our study was fruit and vegetables, which are food substances and have therefore undergone strict controls in order to ensure an adequate health and hygiene profile. 

This aspect concerning the food safety of the substrate was further investigated from a microbiological point of view in different works where the non-presence of pathogenic microorganisms was highlighted [38,39]. Water is another variable in earthworm rearing that may affect safety in addition to the growth substrate. Specifically, in this study, water was a controlled variable because it was potable and was supplied exclusively through diffusers to keep the moisture of the substrate constant.

Considering the volatile profile, the major compound revealed in this SDEM was ethanol as a consequence of the process used to delipidate the sample, as reported by Bou-Maroun and Cayot [21]. The same authors say that delipidation is an important process to increase the organoleptic quality of the raw protein thanks to the reduction of the off-flavour that is described by panelists as a strong animal odor like “dried fish” and to increase stability during long-term storage.

The results of this study showed that some volatile compounds, in particular aldehydes and ketones with hexanal, are present in very low amounts as a direct effect of delipidation. As reported by Cayot et al. [19], the delipidation process leads to the elimination of those organic aromatic compounds which are normally released during lipid oxidation. Hexanal is the principal compound arising from lipid oxidation, and several authors [40,41] have shown that hexanal is the major compound released during the cooking of meat.

Moreover, Romero et al. [42] showed a strong relationship between the amount of extracted lipids and the percentage of dearomatisation. Of The other VOCs found in SDEM, some might stem from the earthworms themselves, the substrate of growth, water and other variables. 

An important role discussed by Montel et al. [43] is the role of bacteria in the production of non-volatile and volatile compounds in the food matrix. These processes are linked to endogenous and microbial enzyme activities, and also to chemical reactions dependent on technological processing; for these reasons the choice of accurately delipidating and sterilizing the earthworm meal was taken in order to keep microbial and aromatic development under control, these two variables being closely related. 

An analysis of recent existing literature showed that few authors [38,44] have deepened food safety aspects related to the meal obtained from earthworms and insects even though they are the basis of a correct “One Health” approach. The results obtained from the microbiological analysis of fresh earthworms, freeze-dried DEM and SDEM samples show significant aspects allowing the identification of a critical point to be controlled in earthworm rearing. The risk analysis carried out on earthworm rearing has led to the choice of different treatment steps in order to prevent hazards and reduce risks. From the microbiological results it is clear that freeze-drying treatment alone cannot guarantee a microbiological profile that complies with food safety requirements. In many contexts earthworm meal is already considered an alternative source of protein in human and animal nutrition and is used in feed supplements for poultry, fish and swine, under certain conditions [45]. Earthworm meal that has not been properly treated remains a nutritional source at risk for humans and animals also considering that transmission routes can be both direct and indirect [45,46]. The most important contamination sources that enter animals through earthworms are antibiotic residues, mycotoxins, pesticides, and bacteria, which can then be transmitted on to humans if the earthworm meal is not properly treated.

As shown by the results obtained in the scientific study, sterilization of the DEM ensures a sterile and, therefore, microbiologically safe end-product. As reported by Byambas et al. [45], some bacteria can develop resistance to conventional heat treatments, e.g., *Staphylococcal enterotoxin* withstands temperatures of 100 °C for 30 min.; the emetic toxin of *B. cereus* resists 90 min. at 126 °C and the botulinum toxin is destroyed at 85 °C for 5 min and after sporulation it should continue for at least 3 min at 121 °C. Time and temperature variables should be carefully considered in order to obtain a safe product to include in human food. On the one hand, in the specific case of steam autoclave sterilization (121 °C for 20 min), it should be considered that this type of treatment could decrease the bioavailability of some amino acids due to protein denaturation [47], and this issue needs further investigation in future work, but the importance and the need to insert a safe food product that therefore respects the parameters of food safety in the food chain is a mandatory and essential prerequisite compared with the preservation of the nutritional and sensory characteristics. On the other hand, in human dietary habits proteins of animal origin are normally subjected to cooking treatment for the protection of consumer health and this treatment involves the reduction of amino acid bioavailability [48,49]. Further studies are necessary to investigate several aspects, from the possible presence of allergens to indirect transmission routes (fresh earthworm–farmed animal–human) since, as reported by Villate [50], some nematodes for whom earthworms are intermediate hosts represent a particular case in the contamination of livestock such as poultry, and this could represent a public health risk for humans.

## 5. Conclusions

This paper aimed to describe a complete series of processing actions in detail for the transfer of pilot earthworm rearing to large-scale production avoiding microbiological risks and any kind of hazard for human health. The results obtained allow it to be concluded that the countless benefits of earthworm rearing include the eco-sustainable management of organic waste from fruit and vegetables. Moreover, this eco-sustainable and ethical solution also provides a valid source of animal protein, addressing future needs for food in a fast-growing population scenario. In conclusion, the fundamental aspect analyzed in this paper and that should be at the basis of all studies focused on the inclusion of this new protein source derived from terrestrial invertebrates in the food chain, is that of food safety with a view to total protection of public health.

## Figures and Tables

**Table 1:** Summary of nutritional, chemical, and microbiological parameters evaluated.

	Investigated Parameters	Reference Methods
**Nutrients**	Dry matter (DM), ash, crude proteins, ether extract	AOAC, 2012. Official Methods of Analysis, 18th ed. Association of Official Analytical Chemists International, Washington, DC, USA.
	Total carbohydrates Water-soluble carbohydrates	Anthrone colorimetric method using UV–Vis spectrophotometer as reported by Deriaz [23].
**Minerals**	Potassium, sodium, calcium, phosphorus, magnesium, iron, aluminium, silicate, chloride, zinc, bromide, manganese, copper, iodine, nickel, barium, chromium, cadmium and lead	Inductive coupled-plasma mass spectrometry (ICP-MS) analyses as reported by Confalonieri et al. [24].
**Pesticides**	Atrazin, azinphos-ethyl, azinphos-methyl, azoxystrobin, benalaxyl, bitertanol, bupirimate, buprofezin, cadusafos, chlorfenvinphos, cyproconazol, cyprodinil, diazinon, ethoprophos, ethoxyquin, fenamiphos, fenarimol, fludioxonil, flusilazole, furalaxyl, kresoxim-methyl, malathion, metalaxyl, methidathion, oxadixyl, paraoxon-methyl, phosalone, piperonyl butoxide, pirimicarb, pirimiphos-ethyl, pirimiphos-methyl, profenophos, propachlor, propargite, pyrazophos, quinalphos, simazine, tetrachlorvinphos, tetraconazole, and triazophos.	Method of analysis as reported by Arioli et al. [25] and Chiesa et al. [26].
**Mycotoxins**	Aflatoxin B1 and B2 and ochratoxin A	Method of analysis as reported by Man et al. [27]
**Antibiotic residues**	Amoxicillin, ampicillin, cloxacillin, dicloxacillin, benzylpenicillin, oxolinic acid, nalidixic acid, cefalexin, cefquinome, ciprofloxacin, enrofloxacin, lomefloxacin, marbofloxacin, florfenicol, florfenicol-amine, chloramphenicol, flumequine, chlortetracycline, doxycycline, oxytetracycline, tetracycline, lincomycin, sulfathiazole, sulfadimidine, sulfadiazine, sulphadimethoxin, trimethoprim, erythromycin, tylosin, thiamphenicol	Method of analysis as reported by Chiesa et al. [28].
**Volatile Organic Compounds (VOCs)**	Alcohols, Aldehydes, Ketones, Sulfur compounds, Free Fatty Acids, Esters, Nitrogen compounds, Hydrocarbons	Method of analysis as reported by Faustini et al. [29].
**Microbiological analyses**	Research of *Salmonella* spp.	UNI EN ISO 6579-1:2017
	Research of *Listeria monocytogenes*	AFNOR BRD 07/04- 09/98
	Enumeration of mesophilic aerobic bacteria	AFNOR 3M 01/1-09/89
	Enumeration of *Enterobacteriaceae*	AFNOR 3M 01/06-09/97
	Enumeration of *E. coli*	AFNOR 3M 01/08-06/01
	Enumeration of total coliforms bacteria	AFNOR 3M 01/2-09/89 A
	Enumeration of coag. + Staphylococci	AFNOR 3M 01/09-04/03 A
	Enumeration of presumed *Bacillus cereus*	UNI EN ISO 7932:2005
	Enumeration of sulphite reducing clostridia	ISO 15213:2003
	Enumeration of spores of sulphite reducing clostridia	

**Table 2:** Quality parameters of the growth substrate.

Parameter	Substrate of Growth
Moisture (%)	84–88
Temperature (°C)	20–25
pH	6.07–8.02
C ^1^ (%DM)	29.5–44
N ^2^ (%DM))	1.3–1.6
C/N	22.7–27.5

^1^ C: carbon, ^2^ N: nitrogen.

**Table 3:** Quality parameters of the sterilized defatted earthworm meal.

Parameter	Sterilized Defatted Earthworm Meal
Ash (%DM)	4.1 ± 0.1
Crude proteins (%DM)	73.2 ± 0.6
Ether extract (%DM)	≤0.1
Total carbohydrates (%DM)	19.5 ± 0.4
Water soluble carbohydrates (% DM)	6.6 ± 0.3
**Minerals (mg kg^−1^)**	
Potassium	4723 ± 46
Sodium	2202 ± 23
Calcium	4014 ± 130
Phosphorus	3082 ± 163
Magnesium	1106 ± 18
Iron	330 ± 22
Aluminium	71 ± 7
Silicate	339 ± 8
Chloride	184 ± 8
Zinc	141 ± 13
Bromide	8 ± 1
Manganese	37 ± 1
Copper	12 ± 1
Iodine	9 ± 1
Nickel	2 ± 0
Barium	ND ^1^
Chromium	4 ± 0
Cadmium	ND ^1^
Lead	ND ^1^

^1^ Not Detected.

**Table 4:** Volatile compound profile of sterilized defatted earthworm meal.

Rt. (min)	Volatile Compounds	ng/g
	**Hydrocarbons**	
1.32	Hexane	9.37
3.84	2.2-dimethyl decane	241.60
8.84	Undecane	184.47
17.95	Tetradecane	92.07
**Tot**		**527.52**
	**Alcohols**	
3.47	Ethanol	9183.74
**Tot**		**9183.74**
	**Free fatty acids**	
21.63	Acetic	85.39
23.69	Propionic	17.31
29.84	Pentanoic	4.52
**Tot**		**107.22**
	**Aldehydes**	
1.46	Acetaldehyde	40.96
2.82	2-methyl butanal	270.24
7.66	Hexanal	35.41
23.2	Benzaldehyde	188.27
**Tot**		**534.88**
	**Ketones**	
2.63	2-butanone	63.06
**Tot**		**63.06**
	**Esters**	
2.01	Acetic acid ethyl ester	11.56
2.53	Acetic acid methyl ester	36.21
3.7	Propanoic acid ethyl ester	33.39
25.14	Dodecanoic acid methyl ester	17.15
**Tot**		**98.31**
	**Sulfur compounds**	
7.35	Dimethyl sulfide	411.27
**Tot**		**411.27**
	**Nitrogen compounds**	
28.52	Formamide	2.24
**Tot**		**2.24**
